# Pre-Natal Exposure to Mouse Parvovirus at Day 5 and 12 Gestation Does Not Induce Immune Tolerance

**DOI:** 10.1371/journal.pone.0156248

**Published:** 2016-05-24

**Authors:** Lon V. Kendall, Celeste Allaband, Kenneth S. Henderson

**Affiliations:** 1 Department of Microbiology, Immunology and Pathology, College of Veterinary Medicine and Biomedical Sciences, Colorado State University, Fort Collins, Colorado, United States of America; 2 Charles River Laboratories International, Wilmington, Massachusetts, United States of America; Rega Institute for Medical Research, BELGIUM

## Abstract

Parvoviruses have a predilection for rapidly dividing cells such as occurs during embryonic development. Potentially, *in utero* exposure could lead to immune tolerance in progeny mice. To determine if MPV infection *in utero* results in immune tolerance, pregnant mice were inoculated by oral gavage with 50 ID_50_ MPV1e or sham inoculated with phosphate buffered saline at day 5 and 12 gestation. Offspring were fostered to MPV-negative recipient dams prior to development of a milk spot. After confirming the offspring were seronegative for MPV by serology and not shedding by fecal PCR, they were challenged with 50 ID_50_ MPV1e by oral gavage at weaning or sham inoculated. At 4 weeks post inoculation, all weanlings exposed *in utero* developed antibodies to MPV, and MPV was detected by fecal PCR. Similarly, all weanlings from sham-inoculated dams challenged with MPV developed antibodies and MPV was detected by fecal PCR. None of the sham inoculated weanling mice from MPV infected dams or sham infected dams developed antibodies to MPV nor was MPV detected by fecal PCR. These results demonstrate that *in utero* exposure to MPV1e via oral gavage was insufficient to induce immune tolerance and provides greater confidence that rederivation techniques may successfully eliminate colonies of MPV. Furthermore, our findings do not provide evidence that MPV tolerance may contribute to hidden infections in mouse colonies.

## Introduction

Mouse parvovirus (MPV) is a commonly recognized infectious agent relatively prevalent in mouse colonies and found in cell culture [1, 2] which can impact research results, such as potentiate rejection of tumor allograft [3]. Elimination of MPV from infected colonies relies principally on rederivation; however, there are aspects of MPV infection that suggest mice may become immune tolerant, for example MPV has been detected in the gametes of mice [4,5]. Since parvoviruses replicate readily in rapidly dividing tissues such as a growing embryo, and gametes may be infected with MPV, vertical transmission of the virus could lead to early *in utero* exposure. There have been recent studies demonstrating rederivation by embryo transfer may not be effective at eliminating minute virus of mice (MVM), a parvovirus similar to MPV. In these studies, fertilized oocytes and morulae were exposed to varying concentrations of MVM. Recipient dams and their progeny receiving embryos infected with as little as 100 ID_50_ seroconverted to MVM. The antibody titers of the progeny dissipated after 20 weeks suggesting they were maternal antibodies, and the authors mentioned unpublished observation that MVM was shed in the experimental colonies for up to one year [6]. One plausible cause for this phenomenon is immune tolerance due to *in utero* exposure.

Within the last several years, we have confirmed MPV seropositivity in three separate immunocompetent mouse breeding rooms, resulting in attempts to eliminate the virus from the colony. On each occasion, at least 100 mice in the colony were tested, and none were identified as seropositive. Although a large representative number of mice were tested, not all mice were tested, so the use of microisolator caging may have sequestered low prevalence breaks within the room, and even within a cage, variation among cage mates could occur. We have also had experiences in which sentinel mice would seroconvert to MPV and subsequent sentinel testing was seronegative for several months, only to be followed by another seropositive sentinel. The cost of confirming or culling colonies due to erratic serological responses can be significant. This erratic serologic detection in sentinels is not uncommon based on conversations with colleagues and presentations at the national meetings. The sporadic nature of the serologic detection of MPV suggested the possibility that the immune response to this pathogen is not consistent with the typical exposure-seroconversion responses noted for most pathogens. The primary rational for pursuing this study was to determine if immune tolerance was responsible for the sporadic serologic responses seen in colony mice during an MPV serologic screen. If parvovirus persists following *in utero* exposure, it could explain the seroconversion of naïve sentinels, but failure to confirm MPV in subsequent serologic screening of colony animals.

For a host to develop an appropriate immune response to pathogens, T cell development occurs in the thymus to make CD4+ or CD8+ T cells. In addition, these cells must undergo positive and negative selection. Positive selection enables T cells to recognize self major histocompatibility complex, while negative selection removes T cells that react to self antigens. The end result is CD4+ and CD8+ T cells capable of recognizing foreign antigens. The mouse thymus begins development at embryonic day 10–12 and is not completely developed until 3–4 weeks after birth. Early experiments with mice have demonstrated one can induce tolerance to foreign antigens if they are introduced in the neonatal period prior to thymic maturation [7]. More recently administration of adeno-associated viral vectors *in utero* and at 2 days post-partum failed to develop detectable antibodies to the viral vector and subsequent challenge with the adeno-associated virus demonstrated tolerance as viral vector expression continued up to 11 months [8]. Immune tolerance due to *in utero* exposure to viral pathogens has been demonstrated with lymphocytic choriomeningitis virus of mice [9], and pestiviruses such as bovine viral diarrhea virus [10] and hog cholera [11]. Similarly, we hypothesized that mice exposed *in utero* develop tolerance to MPV infection resulting in the inability to mount a serologic response following post natal challenge with persistent infection. However, we found that offspring mice exposed *in utero* by oral inoculation of the dam do not develop tolerance to MPV infection.

## Materials and Methods

### Mice

Eighteen female and six male, 10–12 week old outbred Crl:CD1(ICR) mice were purchased from Charles River Laboratories (Wilmington, MA) which were pathogen free for Sendai virus, pneumonia virus of mice, mouse hepatitis virus, minute virus of mice, mouse parvovirus, mouse norovirus, Theiler’s murine encephalitis virus, reovirus, rotavirus, lymphocytic choriomeningitis virus, ectromelia virus, mouse adenovirus, mouse cytomegalovirus, K virus, polyoma virus, Hantavirus, lactate dehydrogenase elevating virus, mouse thymic virus, *Bordetella bronchiseptica*, CAR bacillus, *Citrobacter rodentium*, *Corynebacterium kutcherii*, *Helicobacter species*, *Klebsiella oxytoca*, *Mycoplasma pulmonis*, *Pasteurella pneumotropica*, *Salmonella species*, *Streptobacillus moniliformis*, *Streptococcus pneumonia*, *Clostridium piliforme*, pinworms, and ectoparasites. Mice were housed in individually ventilated caging (Thoren Caging System, Inc, Hazelton, PA). Mice were provided Teklad Irradiated Diet 2918 (Harlan Laboratories, Madison, WI) and filter sterilized water and allowed to acclimate for 7 days prior to initiating the studies. All mice were maintained under a 12:12-h light:dark cycle at temperatures of 21 to 24°C. All animal experiments were approved by the Colorado State University Institutional Animal Care and Use Committee (09-143A-01) and carried out with the recommendations in the *Guide for the Care and Use of Laboratory Animals* of the National Institutes of Health.

### Experimental Infections

Mice were maintained in harems of three females to one male and allowed to breed naturally. Females were checked for vaginal plugs daily to confirm mating. Following successful plugs, female mice were separated. Thirteen time-pregnant female mice were inoculated with 50 ID50 of MPV1e, a mouse passaged strain of MPV, via oral gavage at day 5 and 12 gestation as previously described [12]. Briefly, MPV1e was isolated from a naturally occurring infection and was maintained by oral inoculation of naïve mice with filter-sterilized tissue homogenate. The ID50 was determined in in weaning mice by oral inoculation of serial dilutions [12].Four control time-mated female mice received a control inoculation of 100 microliters of phosphate buffered saline at day 5 and 12 gestation. The pups were fostered to time-mated, MPV negative dams prior to the presence of a milk spot. Once fostered, the original dams were euthanized by asphyxiation with carbon dioxide, blood was collected by cardiocentesis, and feces was collected for PCR analysis.

At 21 days, mice were weaned from the foster dams. Foster dams were euthanized, and blood and tissues collected as described above. Blood from the tail vein and feces were collected from weanling mice to confirm MPV negative status. After weanling mice were confirmed MPV seronegative and PCR negative, 10 male and 10 female mice were orally challenged by gastric gavage with MPV1e, and 5 of each sex received mock inoculum as control, as described above and tested weekly by fecal PCR. Four weeks post-inoculation, mice were euthanized with carbon dioxide. Blood and feces were collected to evaluate viral presence by serology and PCR, respectively.

### Mouse parvovirus Assays

Mice were monitored for fecal shedding by PCR every other day post-inoculation for the first 7 days to confirm MPV infection. Feces was collected and homogenized in PBS as previously described [13], and DNA isolated from fecal pellets using a Qiagen DNA extraction kit according to the manufacturer’s instructions. Quantitative (real time) PCR reactions were performed in a BioRad iCycler using well established primers and cycle times [14].

Serum was collected by centrifugation of blood at 3,000 rpm for 10 minutes and diluted 1:5 in saline. Serologic assays using the Multiplex Fluorometric ImmunoAssay® were performed by Charles River Laboratories using their established protocols to evaluate MPV specific antibodies. Capture antigens used on the Multiplesx Fluorometric ImmunoAssay® platform included the non-structural protein NS-1 and two virus capsid proteins (VP-2) for MPV-1 and MPV-2.

### Statistical Analysis

Analysis was done using SAS 9.4 (SAS Institute, Inc., Cary, NC). Fisher’s exact test was used to compare positive rates (PCR or serology) across inoculation groups (sham/sham, MPV/sham, sham/MPV, MPV/MPV). A separate test was conducted for each testing method and day (PCR- 0, 2, 4, 6, and 28; serology 0 and 28). Similarly, Fisher’s exact test was used to compare conception rates across inoculation groups (sham and MPV). A homoscedastic two-tailed *t*-test was used to compare litter size between MPV and sham infected dams. P-values less than 0.05 were considered statistically significant.

## Results

### Mouse parvovirus infectivity

Of the thirteen pregnant mice inoculated with MPV at day 5 and 12 gestation, eleven of them were confirmed infected by fecal PCR. The foster dams were all fecal PCR negative for MPV. The weanling mice were all PCR and serologically negative to MPV prior to infection. After confirming they were MPV negative, 21 day old weanling mice from MPV inoculated or sham inoculated dams were assigned to two groups for subsequent MPV challenge. Group 1 had 20 mice (10 females, 10 males) from sham inoculated dams that received a sham inoculation; group 2 had 9 mice (5 females, 4 males) from MPV inoculated dams that received a sham inoculation; group 3 had 20 mice (10 females, 10 males) from MPV inoculated dams that received a sham MPV challenge inoculation; and group 4 had 23 mice (11 females, 12 males) from MPV inoculated dams that received an MPV challenge inoculation (S1 Table).

Serum and feces were collected from weanling mice at day 2, 4, 6 and 28 days post inoculation for serology and PCR evaluation. Sham inoculated mice from sham inoculated (Group 1) and MPV inoculated dams (Group 2) were consistently negative at all time points. Fecal PCR results for MPV inoculated mice from sham inoculated dams (Group 3) demonstrated 19 of 20 mice were MPV positive for at least one time point, and fecal PCR assays for MPV inoculated mice from MPV inoculated dams (Group 4) demonstrated 18 of 23 mice were MPV positive for at least one time point. All foster dams were serologically negative to MPV after mice were weaned.

Antibodies to MPV were not detected in the weanling mice originating from either sham or MPV inoculated dams prior to MPV inoculation. At the conclusion of the study, there were no serum antibodies detected in the sham inoculated mice from sham (Group 1) or MPV infected dams (Group 2). Serum antibodies were detected in all MPV inoculated mice from both sham (Group 3) and MPV infected dams (Group 4). Table 1 demonstrates the frequency of positive fecal PCR assays over time for each group and pre- and post-inoculation serology results.

**Table 1:** PCR and serology results from weanling mice pre- and post-MPV inoculation.

			Fecal PCR Results					Serology Results	
		Days PI	0	2	4	6	28	0	28
Group No.	Pregnant Dam Inoculation	Weanling Inoculation							
1	Sham	Sham	0/20	0/20	0/20	0/20	0/20	0/20	0/20
2	MPV	Sham	0/9	0/9	0/9	0/9	0/9	0/9	0/9
3	Sham	MPV	0/20	2/20	14/20*	17/20*	15/20*	0/20	20/20*
4	MPV	MPV	0/23	3/23	8/23*	16/23*	15/23*	0/23	23/23*

Represented as number of positive mice /total number of mice in group.

* represents a statistically significant difference (p < 0.001) compared to the sham/sham group.

### Conception rates and litter size

Seventeen of the 18 mice bred successfully as indicated by the presence of a vaginal plug. Following oral inoculation with MPV1e, five of the 13 MPV infected dams (38%) had pups. All four of the sham inoculated dams (100%) had viable pups. The litter sizes varied greatly between the two groups, although it was not statistically significant (p = 0.08). The litter size of the MPV infected mice ranged from 1–13 with an average litter size of 6.5, while the sham inoculated mice had a litter size ranging from 8–13 with an average of 10 (S1 Data). While this is notable, it was not statistically significant (p = 0.24).

## Discussion

Clearly, our hypothesis that mice exposed *in utero* develop tolerance to MPV infection resulting in the inability to mount a serologic response following post natal challenge with persistent infection was not supported in this study. The mice exposed *in utero* to MPV were able to mount an antibody response to MPV challenge post natal suggesting that oral inoculation of MPV at gestational day 5 and 12 does not result in an immune tolerance in their offspring.

Pregnant mice were inoculated with mouse parvovirus at day 5 and 12 gestation. The initial inoculation of MPV would result in a viremia at approximately 12 days gestation, coinciding with thymic development at embryonic day 10–12 [15]. The subsequent inoculation at day 12 gestation corresponded with thymic development in an effort to maximize thymic exposure to MPV. At this point in thymic development, the T cells would recognize the parvoviral antigens as self, resulting in immune tolerance. This would manifest itself with the inability to mount an antibody response to MPV as an adult, yet continue to have virus persist within the mice. This was not the case in this experiment as all MPV challenged mice seroconverted after exposure *in utero*; therefore, minimizing the chances that immune tolerance is responsible for the sporadic serology responses typically seen in endemic MPV mouse colonies.

There are several studies that indicate that immune tolerance to specific antigens can be induced if the antigen is present during thymic development, including post natal thymic development. In an early experiment demonstrating this phenomenon, CBA mice were inoculated *in utero* with adult tissue cells from an A strain mouse. CBA feti were exposed by a surgical laparotomy and given an intra-embryonic injection of adult tissues from an A strain mouse. The offspring of the CBA mice were subsequently capable of accepting skin grafts from A strain mice, whereas, those that were not exposed *in utero* rejected the skin grafts [7]. The cause for the acceptance of the skin graft from a different mouse strain resulted from immune tolerance induced by early fetal exposure to the antigen. This technique is still used experimentally to overcome the immune response. For example, adeno-associated viral vector used in gene therapy often result in an immune response to the vector and the transgene. To overcome this, immune tolerance was induced in mice via the administration of the protein *in utero* and at day 2 post natal. *In utero* exposure was done by surgically exposing the feti and injecting the antigen intramuscularly. Neonatal exposure was generated via an intramuscular injection at 2 days post natal. In both sets of experiments and routes, challenge with antigen resulted in immune tolerance as demonstrated by a failure to induce an antibody response to the protein [8].

These provide examples of inducing immune tolerance from *in utero* exposure by very invasive means. It is unlikely that this invasive type of exposure would occur in conventional mouse colonies. Because the primary route of infection of MPV is fecal-oral, the oral route of inoculation was chosen to best mimic exposure in a traditional mouse colony. These mice failed to develop immune tolerance with post natal inoculation. Similarly, tolerance was not induced to a viral vector when fetal mice were exposed to the antigen at day 13 to 15 gestation by intramuscular injection [16]. It is certainly possible that the route of inoculation or the concentration of the inoculum was insufficient to induce tolerance; however, the route is typical of what may occur in a conventional mouse facility at a dose that likely exceeds what may be found by the fecal-oral route of exposure. Although every parameter was not investigated, this study does not support that immune tolerance could be an explanation for perceived hidden infections in mouse colonies.

The most common method to eliminate pathogens from mouse colonies is rederivation or embryo transfer, and previous studies have demonstrated successful rederivation of MPV-infected mice by embryo transfer [5]. Since parvoviruses have a predilection for rapidly dividing cells, such as the growing embryo or fetus, one potential complication with this procedure is vertical transmission of pathogens which could result in embryo infection, and consequently immune tolerance. When pregnant mice were challenged with MPV orally in this study, the offspring seroconverted to subsequent MPV challenge after maternal antibodies were found not to be present. Thus the common oral route of transmission in a conventional mouse facility does not result in *in utero* exposure sufficient to result in immune tolerance of the progeny, suggesting vertical transmission is not an important mode of transmission. Therefore rederivation or embryo transfer procedures would be effective in eliminating mouse parvovirus from mouse colonies.

There have been several reports using cross fostering of neonatal mice to eliminate murine pathogens. This includes mouse hepatitis virus, Theiler’s murine encephalomyelitis virus, mouse rotavirus and *Helicobacter hepaticus* [17,18,19]. These studies removed neonates from their dams 24–48 hours after birth and placed them with pathogen free foster dams in a new cage. One study performed an iodine dip of the neonates prior to placing with the foster dam to treat any pathogens on the neonates prior to transfer [19]. When neonates from MPV infected dams were removed prior to developing a milk spot and placed on foster dams in new cages, the mice remained MPV negative by fecal PCR and serology up to 7 weeks of age. Furthermore, their foster dams did not develop antibody titers to MPV suggesting that the pups were not infected while in their original home cage prior to transferring them to the foster dam. While a more extensive study would need to be performed to confirm these observations, it appears that cross fostering may be useful to eliminate MPV from mouse colonies since our study failed to demonstrate *in utero* transmission.

One final observation from this study was the reduced litter size in the MPV infected dams. While there was not a statistically significant difference, the MPV infected dams had reduced conception rates and reduced litter sizes. This may be due to the effects of MPV infection on the developing embryos, similar to the effects human parvovirus may have during pregnancy in people [20].

These results demonstrate that pregnant mice infected with MPV via the oral route do not induce immune tolerance in the offspring. This information bodes well for those using rederivation, embryo transfer or cross fostering as a means to eliminate MPV from their mouse colonies as vertical transmission does not appear to be significant.

## Supporting Information

S1 Data The data set for conception rates and litter sizes. (PDF)

S1 Table The data set for the fecal PCR results and serologic responses. (PDF)

## Abbreviations

MPV: mouse parvovirus
MVM: minute virus of mice
