# Supplementary material for: Pre-Natal Exposure to Mouse Parvovirus at Day 5 and 12 Gestation Does Not Induce Immune Tolerance
Source: PLoS One. 2016 May 24;11(5):e0156248. doi: 10.1371/journal.pone.0156248 (PMC4878799; doi:10.1371/journal.pone.0156248)
Supplement: S1 Data — (PDF) [file pone.0156248.s001.pdf]

Animal ID	Pregnancy	Litter Size
A-MPV	NEG	0
B-MPV	NEG	0
C-MPV	NEG	0
D-MPV	POS	7
E-MPV	NEG	0
F-MPV	NEG	0
G-MPV	POS	1
H-MPV	POS	13
I-MPV	POS	9
J-MPV	NEG	0
K-MPV	NEG	0
L-MPV	POS	3
M-MPV	NEG	0
N-sham	POS	8
O-sham	POS	11
P-sham	POS	13
Q-sham	POS	8
