# Supplementary material for: Pre-Natal Exposure to Mouse Parvovirus at Day 5 and 12 Gestation Does Not Induce Immune Tolerance
Source: PLoS One. 2016 May 24;11(5):e0156248. doi: 10.1371/journal.pone.0156248 (PMC4878799; doi:10.1371/journal.pone.0156248)
Supplement: S1 Table — (PDF) [file pone.0156248.s002.pdf]

Dam MPV status	Weanling Inoculation	Animal	D0	D2	D4	D6	Day 28	Serology D0	Serology D28
sham	sham	WQM-1	NEG	NEG	NEG	NEG	NEG	NEG	NEG
sham	sham	WQM-2	NEG	NEG	NEG	NEG	NEG	NEG	NEG
sham	sham	WQM-3	NEG	NEG	NEG	NEG	NEG	NEG	NEG
sham	sham	WQM-4	NEG	NEG	NEG	NEG	NEG	NEG	NEG
sham	sham	WQM-5	NEG	NEG	NEG	NEG	NEG	NEG	NEG
sham	sham	WNM-1	NEG	NEG	NEG	NEG	NEG	NEG	NEG
sham	sham	WNM-2	NEG	NEG	NEG	NEG	NEG	NEG	NEG
sham	sham	WNM-3	NEG	NEG	NEG	NEG	NEG	NEG	NEG
sham	sham	WNM-4	NEG	NEG	NEG	NEG	NEG	NEG	NEG
sham	sham	WNM-5	NEG	NEG	NEG	NEG	NEG	NEG	NEG
sham	sham	WOF-1	NEG	NEG	NEG	NEG	NEG	NEG	NEG
sham	sham	WOF-2	NEG	NEG	NEG	NEG	NEG	NEG	NEG
sham	sham	WOF-3	NEG	NEG	NEG	NEG	NEG	NEG	NEG
sham	sham	WOF-4	NEG	NEG	NEG	NEG	NEG	NEG	NEG
sham	sham	WOF-5	NEG	NEG	NEG	NEG	NEG	NEG	NEG
sham	sham	WPF1-1	NEG	NEG	NEG	NEG	NEG	NEG	NEG
sham	sham	WPF1-2	NEG	NEG	NEG	NEG	NEG	NEG	NEG
sham	sham	WPF1-3	NEG	NEG	NEG	NEG	NEG	NEG	NEG
sham	sham	WPF1-4	NEG	NEG	NEG	NEG	NEG	NEG	NEG
sham	sham	WPF1-5	NEG	NEG	NEG	NEG	NEG	NEG	NEG
MPV	sham	WHM1-1	NEG	NEG	NEG	NEG	NEG	NEG	NEG
MPV	sham	WHM1-2	NEG	NEG	NEG	NEG	NEG	NEG	NEG
MPV	sham	WHM1-3	NEG	NEG	NEG	NEG	NEG	NEG	NEG
MPV	sham	WHM1-4	NEG	NEG	NEG	NEG	NEG	NEG	NEG
MPV	sham	WHF-1	NEG	NEG	NEG	NEG	NEG	NEG	NEG
MPV	sham	WHF-2	NEG	NEG	NEG	NEG	NEG	NEG	NEG
MPV	sham	WHF-3	NEG	NEG	NEG	NEG	NEG	NEG	NEG
MPV	sham	WHF-4	NEG	NEG	NEG	NEG	NEG	NEG	NEG
MPV	sham	WHF-5	NEG	NEG	NEG	NEG	NEG	NEG	NEG
sham	MPV	WQF2-1	NEG	NEG	POS	POS	POS	NEG	POS
sham	MPV	WQF2-2	NEG	NEG	POS	POS	POS	NEG	POS
sham	MPV	WQF2-3	NEG	NEG	POS	POS	NEG	NEG	POS
sham	MPV	WPF2-1	NEG	NEG	POS	POS	POS	NEG	POS
sham	MPV	WPF2-2	NEG	NEG	NEG	NEG	POS	NEG	POS
sham	MPV	WPF2-3	NEG	NEG	NEG	POS	NEG	NEG	POS
sham	MPV	WPF2-4	NEG	NEG	NEG	POS	POS	NEG	POS
sham	MPV	WNF2-1	NEG	NEG	POS	POS	POS	NEG	POS
sham	MPV	WNF2-2	NEG	NEG	POS	POS	POS	NEG	POS
sham	MPV	WNF2-3	NEG	POS	POS	POS	POS	NEG	POS
sham	MPV	WOM2-1	NEG	NEG	NEG	POS	POS	NEG	POS
sham	MPV	WOM2-2	NEG	NEG	POS	NEG	NEG	NEG	POS
sham	MPV	WOM2-3	NEG	NEG	POS	POS	POS	NEG	POS
sham	MPV	WOM1-1	NEG	POS	POS	POS	POS	NEG	POS
sham	MPV	WOM1-2	NEG	NEG	NEG	POS	POS	NEG	POS
sham	MPV	WOM1-3	NEG	NEG	POS	POS	POS	NEG	POS
sham	MPV	WPM-1	NEG	NEG	POS	POS	POS	NEG	POS
sham	MPV	WPM-2	NEG	NEG	NEG	NEG	NEG	NEG	POS
sham	MPV	WPM-3	NEG	NEG	POS	POS	POS	NEG	POS
sham	MPV	WPM-4	NEG	NEG	POS	POS	NEG	NEG	POS
MPV	MPV	WLF-1	NEG	NEG	NEG	NEG	POS	NEG	POS
MPV	MPV	WLF-2	NEG	NEG	NEG	POS	POS	NEG	POS
MPV	MPV	WLF-3	NEG	NEG	NEG	POS	POS	NEG	POS
MPV	MPV	WIF-1	NEG	NEG	POS	POS	NEG	NEG	POS
MPV	MPV	WIF-2	NEG	NEG	POS	POS	POS	NEG	POS
MPV	MPV	WIF-3	NEG	NEG	NEG	POS	NEG	NEG	POS
MPV	MPV	WIF-4	NEG	POS	NEG	NEG	POS	NEG	POS
MPV	MPV	WIM-1	NEG	NEG	NEG	POS	POS	NEG	POS
MPV	MPV	WIM-2	NEG	POS	NEG	POS	POS	NEG	POS
MPV	MPV	WIM-3	NEG	NEG	POS	POS	POS	NEG	POS
MPV	MPV	WIM-4	NEG	NEG	POS	POS	POS	NEG	POS
MPV	MPV	WIM-5	NEG	NEG	POS	POS	POS	NEG	POS
MPV	MPV	WHM2-1	NEG	NEG	NEG	POS	POS	NEG	POS
MPV	MPV	WHM2-2	NEG	POS	POS	POS	POS	NEG	POS
MPV	MPV	WHM2-3	NEG	NEG	NEG	POS	POS	NEG	POS
MPV	MPV	WHM2-4	NEG	NEG	NEG	POS	POS	NEG	POS
MPV	MPV	WDM-1	NEG	NEG	NEG	NEG	NEG	NEG	POS
MPV	MPV	WDM-2	NEG	NEG	NEG	NEG	NEG	NEG	POS
MPV	MPV	WDM-3	NEG	NEG	POS	POS	POS	NEG	POS
MPV	MPV	WDF-1	NEG	NEG	NEG	NEG	NEG	NEG	POS
MPV	MPV	WDF-2	NEG	NEG	NEG	NEG	NEG	NEG	POS
MPV	MPV	WDF-3	NEG	NEG	NEG	NEG	NEG	NEG	POS
MPV	MPV	WDF-4	NEG	NEG	POS	POS	NEG	NEG	POS
